# Raddeanin A inhibits proliferation, invasion, migration and promotes apoptosis of cervical cancer cells via regulating miR-224-3p/Slit2/Robo1 signaling pathway

**DOI:** 10.18632/aging.202574

**Published:** 2021-02-17

**Authors:** Xin Shen, Lingxia Li, Yuanyuan He, Xiaohui Lv, Jiajia Ma

**Affiliations:** 1 Department of Gastrointestinal Surgery, Xi’an Daxing Hospital, Xi’an 71000, Shannxi Province, China; 2 Department of Obstetrics and Gynecology, Fourth Military Medical University, Xi’an 710032, Shannxi Province, China

**Keywords:** cervical cancer, Raddeanin A, proliferation, invasion, migration

## Abstract

Raddeanin A (RA), an active triterpenoid saponin extracted from the *Anemone raddeana* regel, plays an essential role in the suppression of many malignancies. We aimed to investigate the effects and potential mechanisms of RA on cervical cancer (CC). RA was used to treat CC cell lines (Hela and c-33A) for 24 h and 48 h. Then, the invasion, migration and cell cycle distribution of these two cell lines with RA treatment were respectively detected by transwell, wound healing and flow cytometry. Results revealed that RA significantly inhibited the invasion, migration, promoted the cell cycle arrest and apoptosis of Hela and c-33A cells. Moreover, RA was confirmed to activate the Slit2/Robo1 signaling, and bioinformatics analysis and luciferase reporter assay verified that miR-224-3p could target Slit2. Additionally, miR-224-3p overexpression reversed the inhibitory effect of RA on invasion and migration of CC cells, and it also restored the promoting effects of RA on cell cycle arrest and apoptosis. Lastly, miR-224-3p-upregulation inactivated the expression of Slit2 and Robo1 in RA-treated Hela and c-33A cells. These findings demonstrated that RA inhibits proliferation, invasion, migration and promotes apoptosis of CC cells through miR-224-3p/Slit2/Robo1 signaling pathway, which might guide the future studies or treatment of this disease.

## INTRODUCTION

Cervical cancer (CC) is the third most common gynecologic tumor that poses an enormous threat to women’s health [1]. The number for new CC cases is reported to reach 528,000 and 266,000 people will die from this disease every year. Moreover, the mortality rate in countries with low income or resources is almost three times higher than those with high income or resources [2, 3]. Treatments for mid-term and advanced CC appear to be limited with little effects. CC patients are confronted with low survival rate and terror as the survival rate for patients with or without treatment has been decreasing sharply in recent years [4–6]. Thus, finding a therapeutically effective drug is of great importance for CC treatment.

Raddeanin A (RA) is a primary triterpenoid saponin extracted from Ranunculaceae *Anemone raddeana* rhizome. It is a common Chinese traditional medicinal agent used to promote diuresis [7]. In recent years, RA has been reported to inhibit tumor growth, including gastric cancer, breast cancer and osteosarcoma [8–11]. A previous study demonstrated that RA could promote gastric cancer cell apoptosis and autophagy by activating p38 MAPK pathway [12]. Additionally, compelling evidence indicated that RA could inhibit the growth of colorectal cancer cell through downregulating Wnt/β catenin signaling and NF-κB pathway [13]. Therefore, RA seems to be an effective modulator for tumor growth and progression. A rodent experiment indicated that RA has an ideal anti-tumor activity to suppress CC growth, but the specific molecular mechanism remains unclear [8].

MicroRNAs (miRNAs) are a series of non-coding RNAs involved in posttranscriptional gene expression. Their regulation on tumor development including differentiation, proliferation, angiogenesis, invasion, and metastasis has been widely reported [14, 15]. MiR-224-3p, as one member of miRNAs, was found to be closely related to human papillomavirus (hrHPV) infection and CC progression [16, 17]. Slit guidance ligand 2 (Slit2), a tumor suppressor gene, was found to play diverse roles in apoptosis, neurogenesis and angiogenesis of many malignancies by binding to its receptor Robo1 and then transducing the intracellular signaling [18]. Emerging evidence supports that the inactivation of Slit2/Robo1 signaling pathway is of great significance for the control of CC development [19]. Consequently, we infer that RA may suppress CC progression through miR-224-3p/Slit2/Robo1 signaling pathway.

In this study, we observed how RA affected CC cell (Hela and c-33A) proliferation, invasion, migration, cell cycle and apoptosis and explored its mechanism related to miR-224-3p/Slit2/Robo1 signaling pathway. This study potentially provides a theoretical and experimental basis for the exploration of a novel method to treat CC.

## RESULTS

### RA decreases the cell viability of CC cells

**Figure 1:**
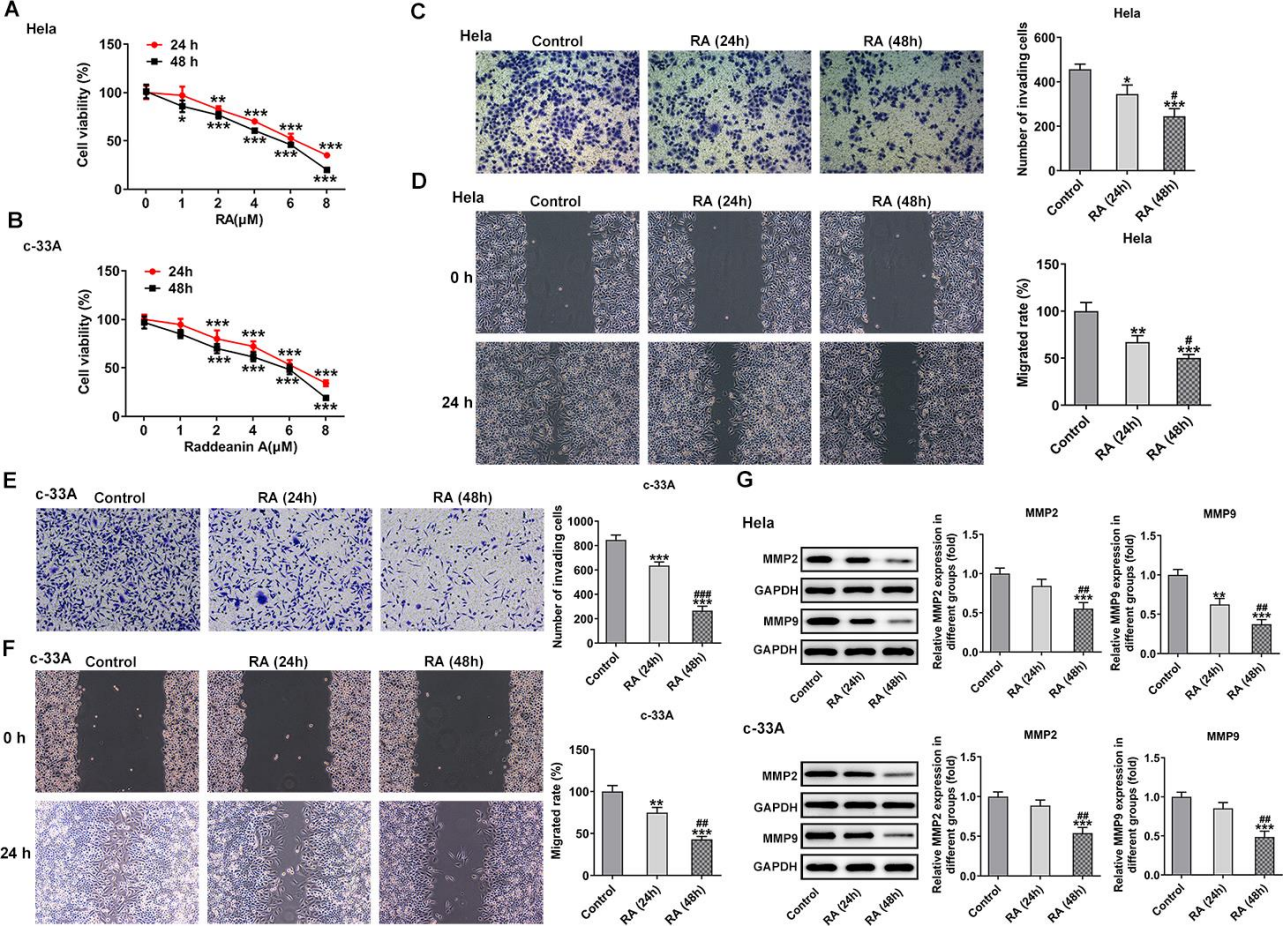
**RA decreases the cell viability and inhibits the invasion and migration of CC cells.** CCK-8 assay was used to detect the cell viability of (**A**) Hela and (**B**) c-33A cells treated with RA. ^*^P<0.05, ^*^P<0.01, ^*^P<0.001 vs. untreated group. (**C**) Transwell and (**D**) wound healing assays were respectively conducted for the detection of invasion and migration of Hela cells. (**E**) Transwell and (**F**) wound healing assays were employed to examine the invasion and migration of c-33A cells, respectively. (**G**) Western blot analysis was used to detect the expression of MMP-2 and MMP-9 in both Hela and c-33A cells. ^*^P<0.05, ^*^P<0.01, ^*^P<0.001 vs. control; ^#^P<0.05, ^#^P<0.01, ^#^P<0.001 vs. RA (24 h).

To examine the function of RA on CC cells, two human CC cell lines, which were Hela and c-33A, were used for the experiments in this study. The cells were treated with different concentrations of RA (0, 1, 2, 4, 6, and 8 μM) for 24 h and 48 h, respectively. Subsequently, cells were detected by CCK-8 assay to assess the cell viability. As shown in Figure 1A, 1B, cell viability was significantly reduced as RA concentration increased, which was in consistent with the previous studies [11, 20]. At last, we chose 4 μM of RA for the following experiments as these cells treated with 4 μM of RA showed relatively moderate cell viability.

### RA inhibits the invasion and migration of CC cells

CC cell invasion and migratory abilities were evaluated by using transwell assay and wound healing assay after Hela and c-33A cells were treated with 4 μM RA for 24 h and 48 h. Results in Figure 1C–1F exhibited that the abilities of invasion and migration of Hela and c-33A cells were conspicuously reduced by RA in a time-dependent manner as comparison to the control group. In addition, results from western blotting indicated the expression of MMP-2 and MMP-9 proteins, which are great contributors to tumor cell migration, was significantly downregulated after the induction of RA in these two CC cell lines (Figure 1G). These results suggest that RA notably inhibits the invasion and migration of CC cells.

### RA promotes the cell cycle arrest and cell apoptosis of CC cells

**Figure 2:**
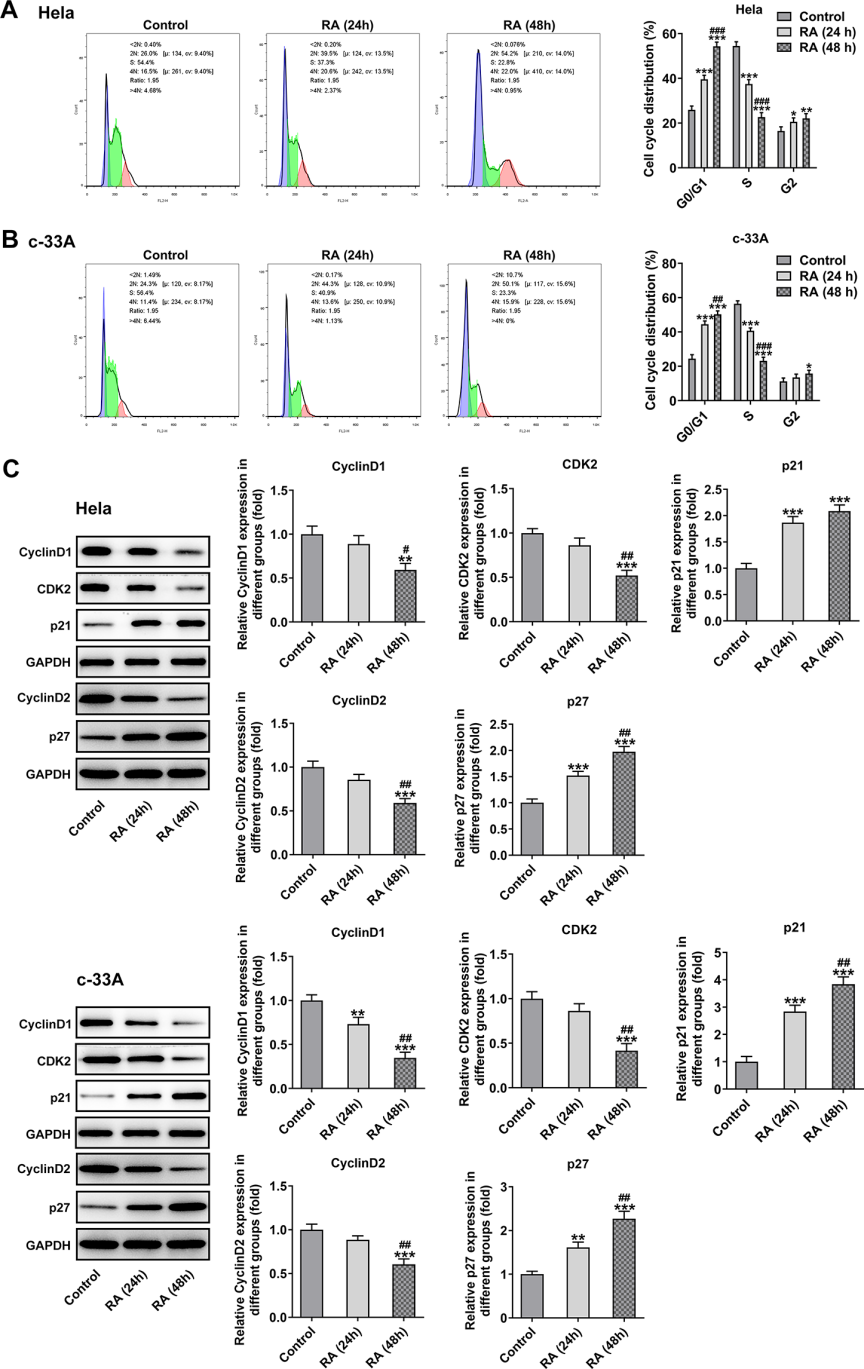
**RA promotes cell cycle arrest of CC cells.** Flow cytometry was used for the detection of cell cycle in (**A**) Hela and (**B**) c-33A cells treated with RA. (**C**) The expression of cell cycle-related proteins in Hela and c-33A cells was determined using western blot analysis. ^*^P<0.05, ^*^P<0.01, ^*^P<0.001 vs. control; ^#^P<0.05, ^#^P<0.01, ^#^P<0.001 vs. RA (24 h).

**Figure 3:**
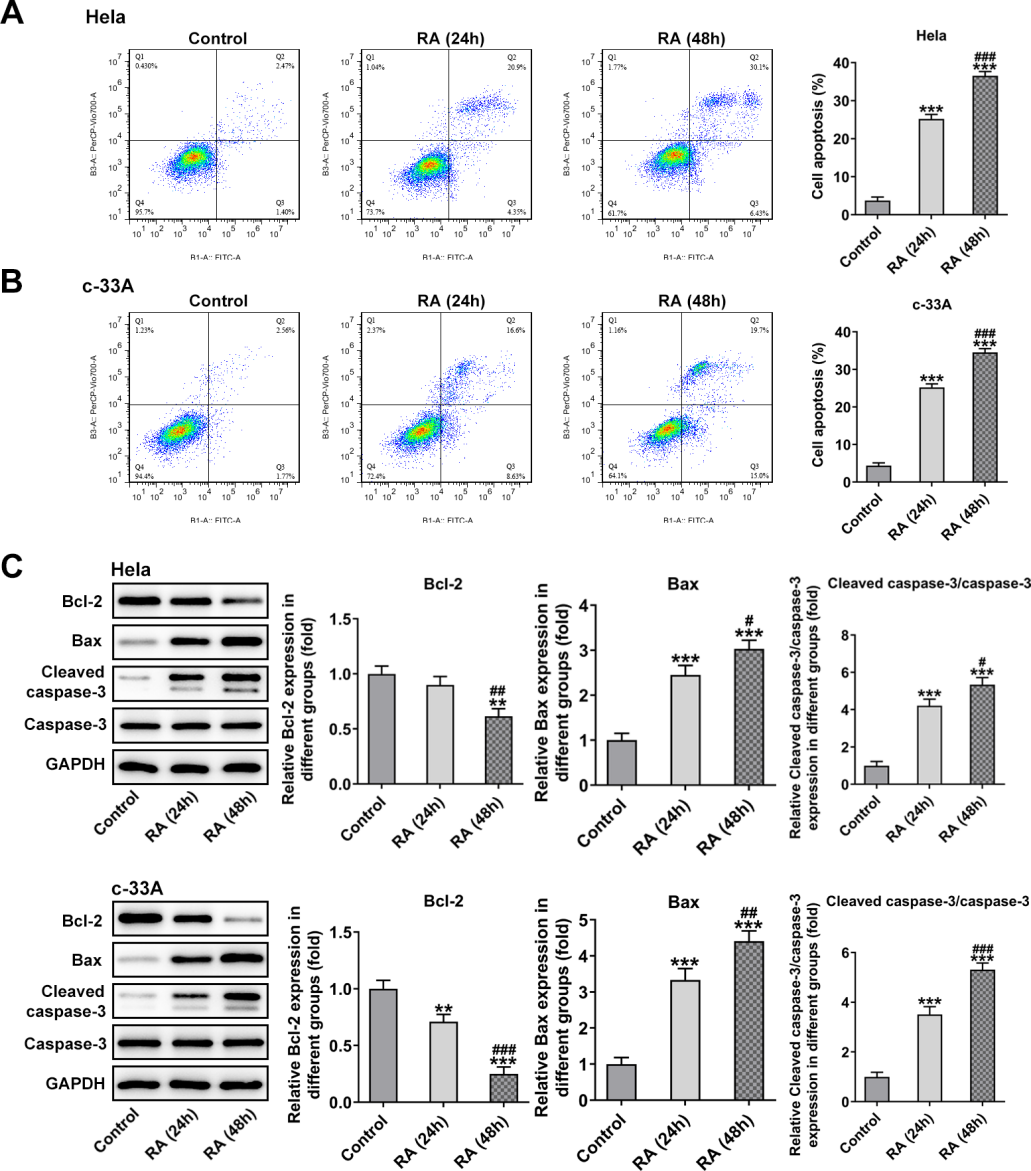
**RA promotes cell apoptosis of both Hela and c-33A cells.** Flow cytometry and was conducted for determination the apoptosis of (**A**) Hela and (**B**) c-33A cells. (**C**) The expression of apoptosis-related proteins in both Hela and c-33A cells was assess using western blot analysis. ^*^P<0.01, ^*^P<0.001 vs. control; ^#^P<0.05, ^#^P<0.01, ^#^P<0.001 vs. RA (24 h).

It has been confirmed that the endless proliferation of tumor cells was due to cell cycle disorder and lack of apoptosis mechanism [21]. Thus, the cell cycle and apoptosis of CC cells were respectively detected by means of flow cytometry analysis. The cell cycle with RA doses exhibited an increase in G0/G1 phase and a decrease in S phase relative to the control group in Hela (Figure 2A) and c-33A (Figure 2B) cells. Concurrently, the decreased levels of cell cycle-related proteins, cyclinD1, cyclinD1 and CDK2, and increased level of p21 and p27 also indicated the promoting role of RA in cell cycle arrest (Figure 2C). As it is observable from Figure 3A, the number of apoptotic Hela cells was notably increased after RA treatment relative to the untreated control group. And RA intervention for 48 h presented the higher apoptotic rate than 24 h. As expected, the same changing trend of apoptotic rate in c-33A cells following RA treatment was observed in Figure 3B. Additionally, as displayed in Figure 3C, RA exposure markedly downregulated the expression of anti-apoptotic protein Bcl-2 and upregulated that of pro-apoptotic proteins Bax and cleaved caspase-3, suggesting that RA promotes the CC cell apoptosis. Taken together, these data provide evidence that RA can induce the cell cycle arrest and promote apoptosis in both Hela and c-33A cells.

### RA activates the Slit2/Robo1 signaling pathway

**Figure 4:**
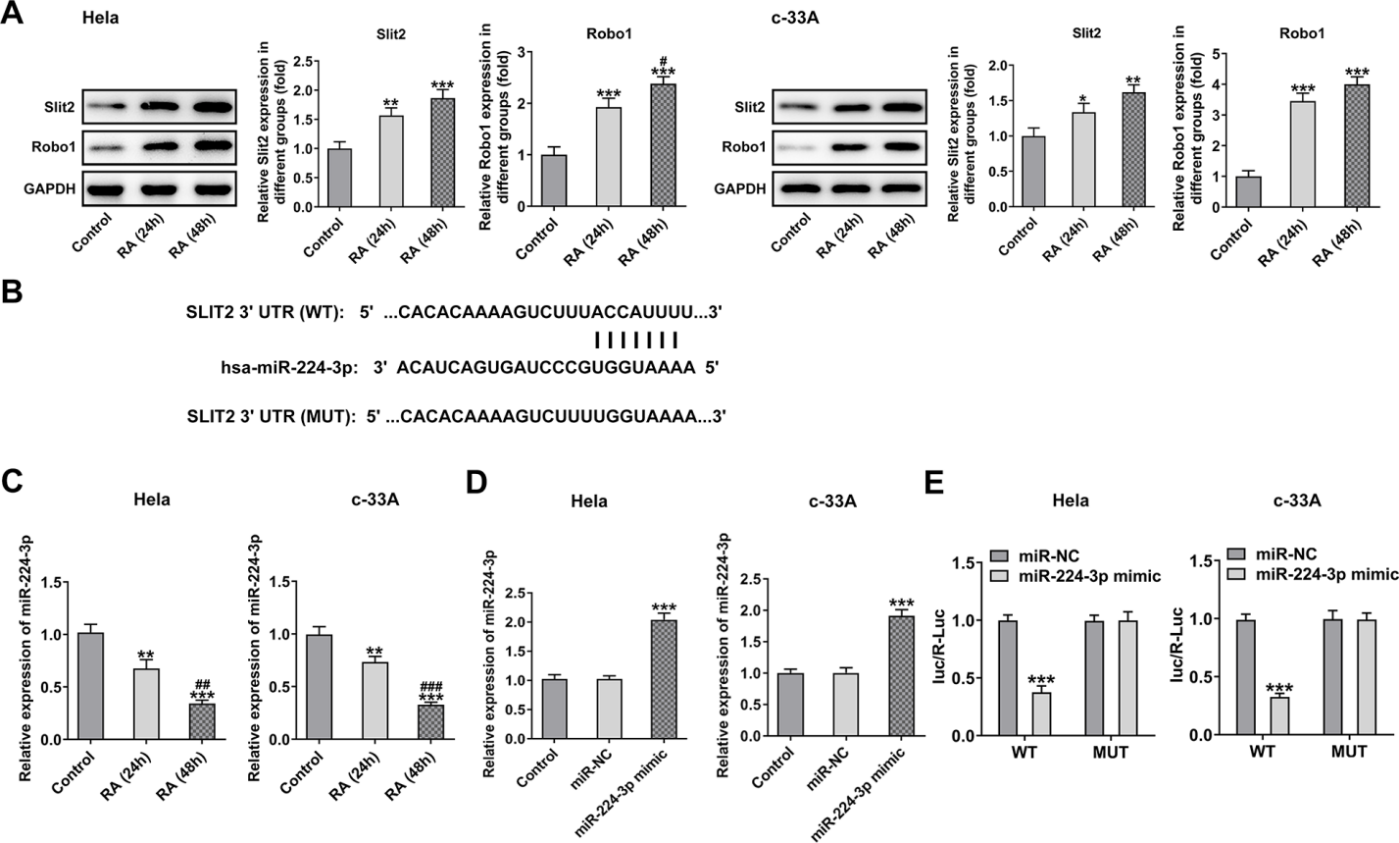
**RA activates the Slit2-Robo1 signaling pathway.** (**A**) The expression of Slit2 and Robo1 was detected by western blot analysis in Hela and c-33A cells treated with RA. ^*^P<0.05, ^*^P<0.01, ^*^P<0.001 vs. control; ^#^P<0.05 vs. RA (24 h). (**B**) The binding sites between miR-224 and Slit2 by bioinformatics analysis. (**C**) The expression of miR-224-3p after RA treatment was detected by RT-qPCR in these two cell lines. ^*^P<0.01, ^*^P<0.001 vs. control; ^#^P<0.01, ^#^P<0.001 vs. RA (24 h). (**D**) The expression of miR-224-3p was tested using RT-qPCR after transfection with miR-224-3p mimic in Hela and c-33A cells. ^*^P<0.001 vs. miR-NC. (**E**) The luciferase reporter assays were used to verify the binding sites between miR-224-3p and Slit2. ^*^P<0.001 vs. miR-NC.

A recent study has indicated that Slit2 was low expressed in Hela cells, and it might be a vital anti-tumor gene [19]. The Slit2/Robo1 signaling pathway was reported to participate in the tumor progression of CC [22]. Therefore, this signaling was tested in RA-induced CC cells by western blot analysis to evaluate whether RA can regulate it. The results revealed that the levels of Slit2 and Robo1 were remarkably increased after RA treatment for 24 h and 48 h compared with the control group in both Hela and c-33A (Figure 4A) cells. Subsequently, by bioformatic analysis, miRDB predicated the target binding between miR-224-3p and Slit2, which was exhibited in Figure 4B. As detected by RT-qPCR, the mRNA level of miR-224-3p was notably downregulated in Hela and c-33A cells induced by RA relative to the control group (Figure 4C). Additionally, the expression of miR-224-3p was markedly upregulated after transfection with miR-224-3p mimic (Figure 4D). Subsequently, the luciferase reporter assay displayed that the luciferase activity of WT-Slit2+miR-224-3p mimic group was weaker than MUT-Slit2+miR-224-3p mimic (Figure 4E). Therefore, it can be inferred that RA may facilitate CC proliferation, invasion and metastasis through miR-224-3p/Slit2/Robo1 signaling pathway.

### Overexpression of miR-224-3p attenuates the inhibitory effect of RA on CC cell proliferation, invasion and migration

**Figure 5:**
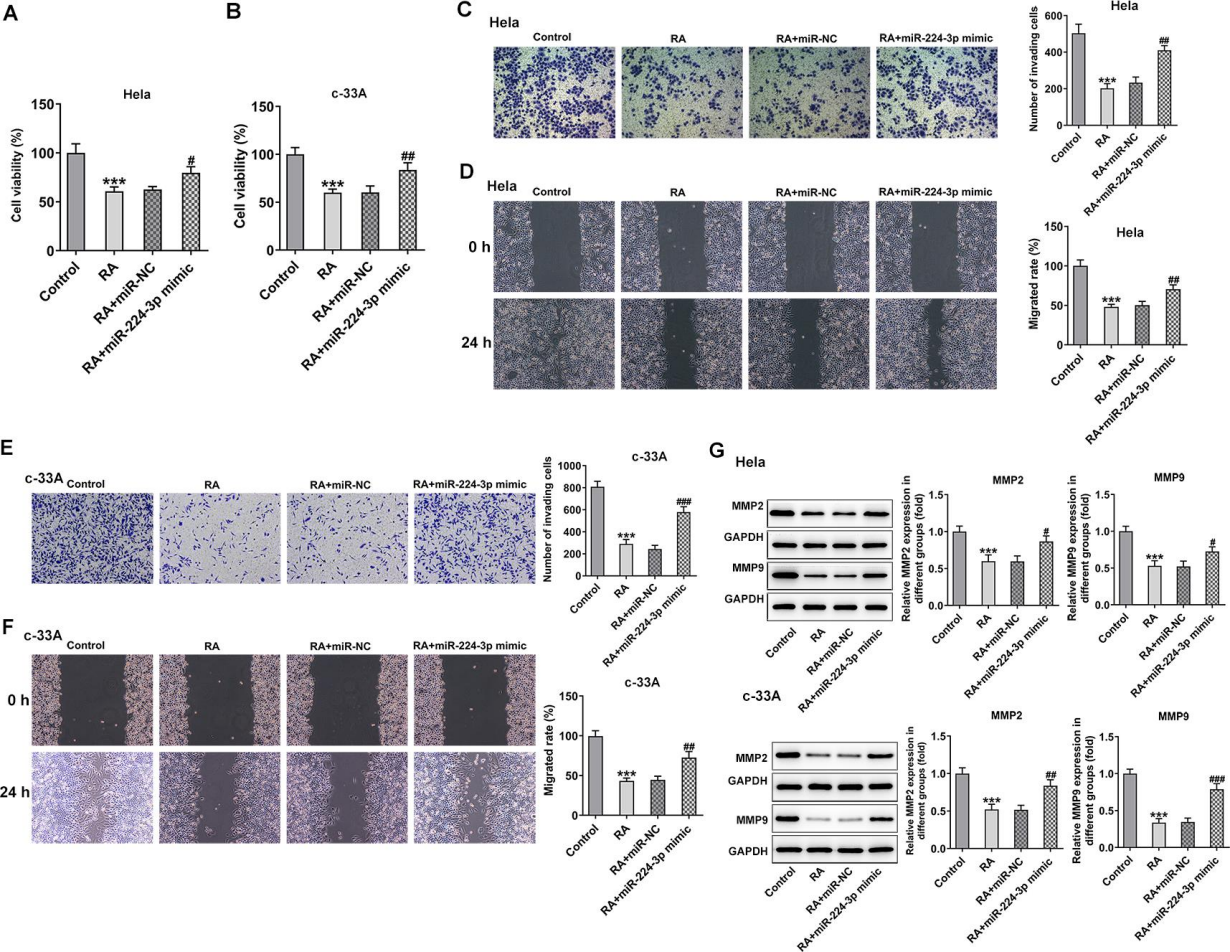
**Overexpression of miR-224-3p reverses the inhibitory effect of RA on CC cell invasion and migration.** The proliferation of (**A**) Hela and (**B**) c-33A cells was detected by CCK-8 assay. (**C**) Transwell and (**D**) wound healing assays were respectively conducted for the detection of invasion and migration of Hela cells. (**E**) Transwell and (**F**) wound healing assays were employed to examine the invasion and migration of c-33A cells, respectively. (**G**) The expression of MMP-2 and MMP-9 in Hela and c-33A cells was evaluated by western blot analysis. ^*^P<0.001 vs. control; ^#^P<0.05, ^#^P<0.01, ^#^P<0.001 vs. RA+miR-NC.

To further clarify the regulatory mechanism of RA in the progression of CC, miR-224-3p mimic was transfected into both Hela and c-33A cells. Then, CCK-8 assay was used to assess the effect of miR-224-3p overexpression on CC cell proliferation. As presented in Figure 5A, 5B, RA significantly reduced the cell proliferation of Hela and c-33A cells, while miR-224 mimic reversed this effect. Moreover, the results from transwell and wound healing assays indicated that the inhibitory effects of RA on invasion and migration of Hela (Figure 5C, 5D) and c-33A (Figure 5E, 5F) cells were dramatically alleviated by miR-224-3p mimic. Consistently, the expression of MMP-2 and MMP-9 in Hela and c-33A cells was markedly downregulated upon RA treatment while upregulated after transfection with miR-224-3p mimic (Figure 5G). Thus, overexpression of miR-224-3p reverses the inhibitory effect of RA on CC cell invasion and migration.

### Overexpression of miR-224-3p reverses cell cycle arrest and CC cell apoptosis induced by RA

**Figure 6:**
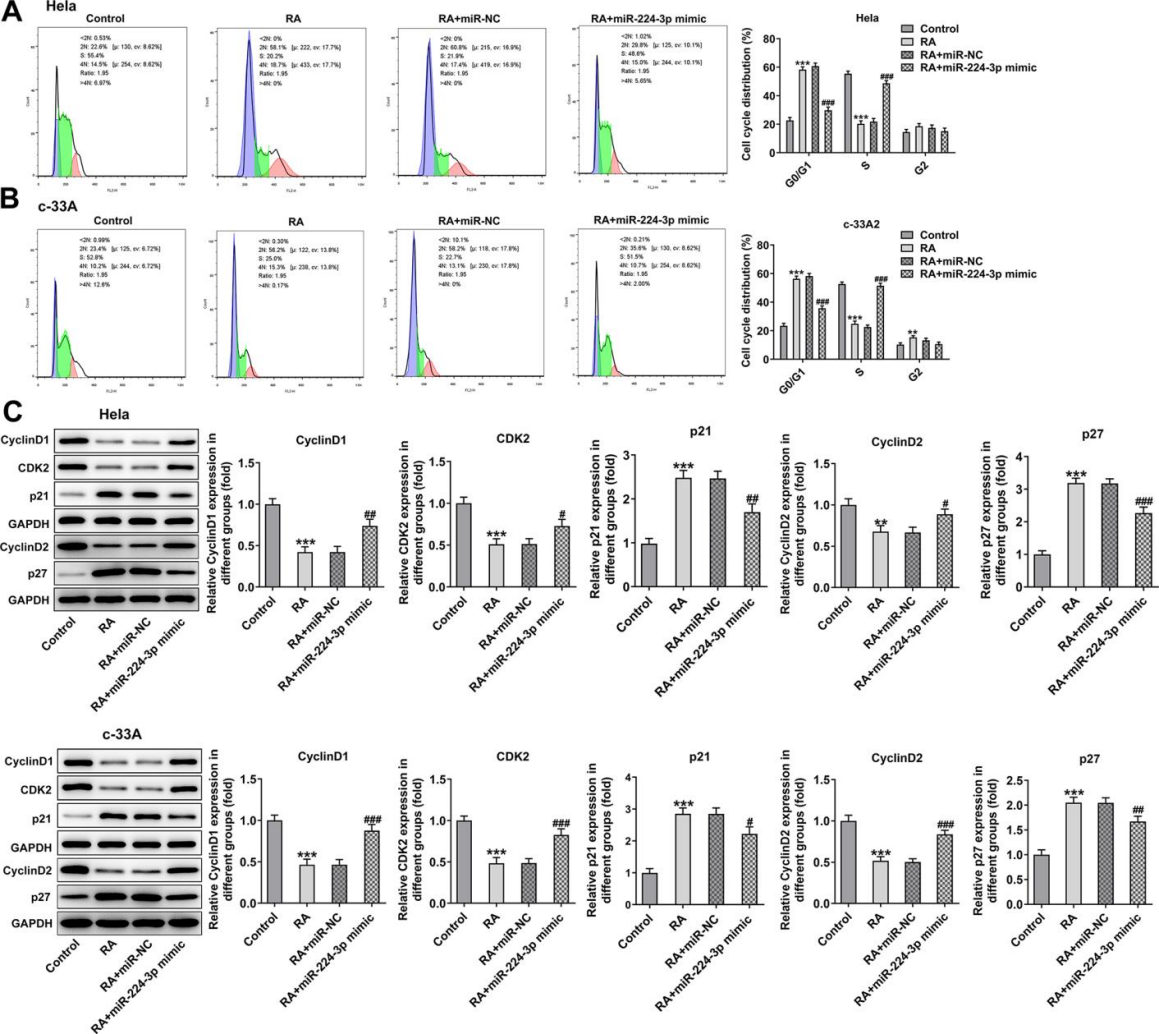
**Overexpression of miR-224-3p attenuates the cell cycle arrest of CC cells induced by RA.** Flow cytometry was used for the detection of cell cycle in (**A**) Hela and (**B**) c-33A cells treated with RA. (**C**) Western blot analysis was used to detect the expression of cell cycle-related proteins in both Hela and c-33A cells. ^*^P<0.01, ^*^P<0.001 vs. control; ^#^P<0.05, ^#^P<0.01, ^#^P<0.001 vs. RA+miR-NC.

With respect to cell cycle and cell apoptosis, results in Figure 6A, 6B displayed the drastic decline in S phase and promotion in G0/G1 phase induced by RA in Hela and c-33A cells were reversed by miR-224-3p mimic.

**Figure 7:**
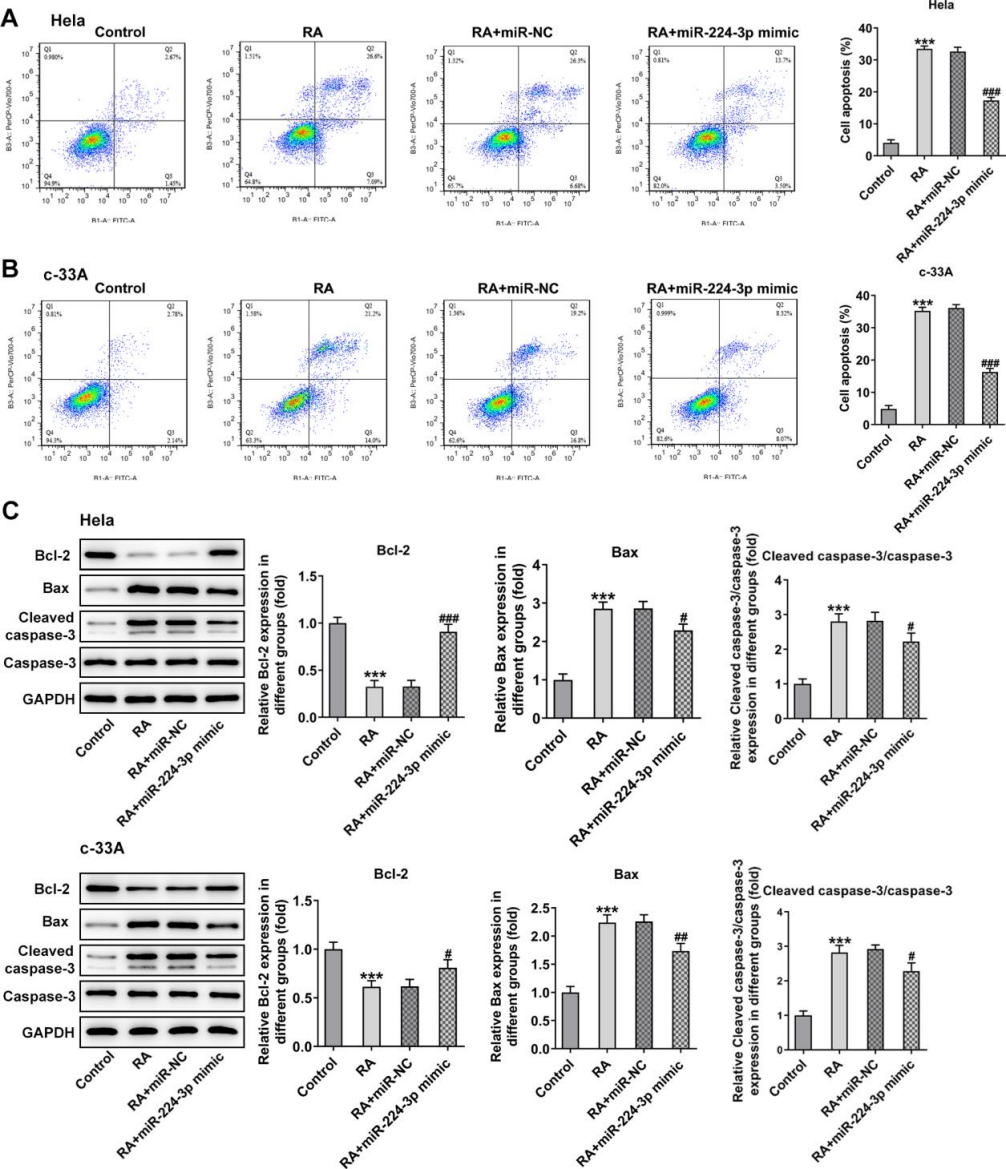
**Overexpression of miR-224-3p reverses the cell apoptosis of CC cells induced by RA.** Flow cytometry and was conducted for determination the apoptosis of (**A**) Hela and (**B**) c-33A cells. (**C**) The expression of apoptosis-related proteins in Hela and c-33A cells was assess using western blot analysis. ^*^P<0.001 vs. control; ^#^P<0.05, ^#^P<0.01, ^#^P<0.001 vs. RA+miR-NC.

Consistently, the expression of cyclinD1, cyclinD2 and CDK2 was notably upregulated while that of p21 and p27 was downregulated in the RA-treated Hela and c-33A (Figure 6C) cells with miR-224-3p overexpression. Additionally, as exhibited in Figure 7A, Hela cells transfected with miR-224-3p mimic possessed the lower apoptotic rate than that transfected with miR-NC. Meanwhile, the number of apoptotic c-33A cells presented the same results with Hela cells (Figure 7B). Moreover, as it is observable from Figure 7C, RA reduced the expression of anti-apoptotic protein Bcl-2 and elevated that of pro-apoptotic proteins Bax and cleaved caspase-3, but miR-224-3p mimic triggered the increase in expression of Bcl-2 and the decrease in that of Bax and cleaved caspase-3 in both Hela and c-33A cells. Collectively, these findings provide a clue that miR-224-3p overexpression partly blocks cell cycle arrest and CC cell apoptosis induced by RA in CC cells.

### Overexpression of miR-224-3p mitigates the Slit2/Robo1 signaling pathway in RA-treated CC cells

**Figure 8:**
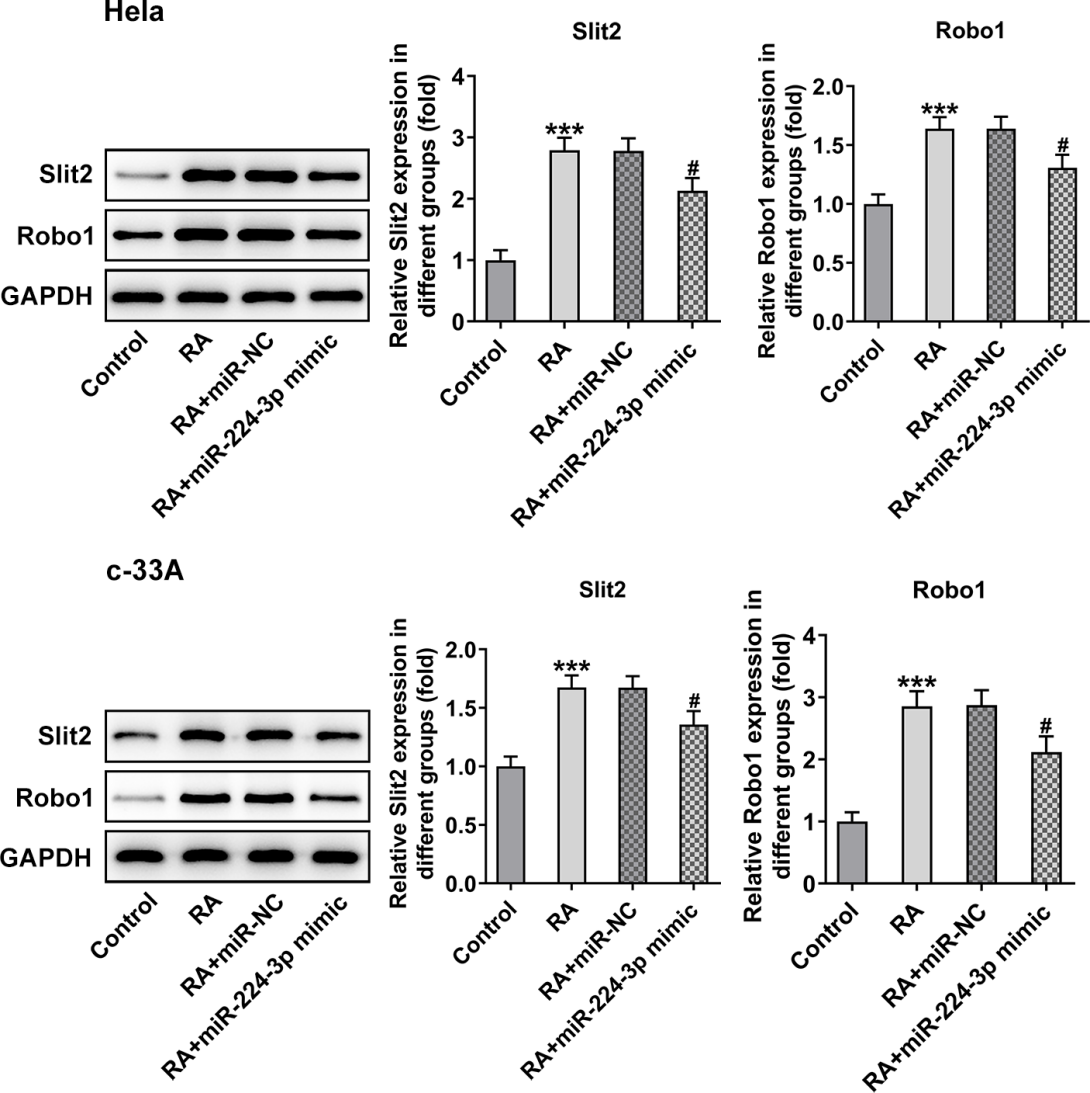
**Overexpression of miR-224-3p restores the expression of Slit2 and Robo1 in CC cells induced by RA.** The expression of Slit2 and Robo1 in Hela and c-33A cells was detected by western blot analysis. ^*^P<0.001 vs. control; ^#^P<0.001 vs. RA+miR-NC.

Then, the effect of miR-224-3p on the Slit2/Robo1 signaling pathway was detected by western blot analysis. As displayed in Figure 8, the expression levels of Slit2 and Robo1 were remarkably elevated upon RA treatment relative to the control group. However, as comparison to the miR-NC group, the miR-224-3p overexpression group exhibited notably downregulated expression of Slit2 and Robo1, suggesting that the overexpression of miR-224-3p inactivates the Slit2/Robo1 signaling pathway in RA-treated CC cells.

## DISCUSSION

CC is one of the most common cancers in gynecologic carcinomas that bring a great burden to the families and the society, especially in developing countries [23]. The prognosis of intermediate and advanced stage CC remains poor, with a low median survival of 8-13 months [24]. To date, CC still represents a difficult clinical challenge as a result of atypical symptoms of CC. Facing a situation where clinical treatments for advanced CC present limitations and the unclear molecular mechanism of CC, efficient therapies are increasingly and urgently needed.

Due to the side effects of chemotherapy and radiography, the use of traditional Chinese medicine has become widely accepted as an effective method to treat cancers. Being an oleanane-type triterpenoid saponin extracted from the root of *Anemone raddeana* Regel, RA displays a promising anti-tumor effect on malignant tumors, including endometrial cancer and prostate cancer [25, 26]. Compelling evidence indicates that RA inhibits the invasion, migration, adhesion and induces apoptosis of human gastric cancer cells, exhibiting potential to become an antitumor drug [9]. Importantly, the same effects RA exerts is found in colorectal cancer [20]. For the first time, the present study explored the effects and potential mechanisms of RA in both Hela and c-33A cells. And we demonstrated that RA treatment potently suppressed the invasion and migration, and promoted the cell cycle arrest and apoptosis of CC cells.

As a member of the Slit family, Slit2 is the ligand of Robo1 receptor to promote organ development and show pro-angiogenic abilities [27, 28]. The expression of Slit2 was low in Hela cells, and it was speculated to be an important tumor suppressor [19]. Various cellular processes, including cell-cell adhesion and invasion, can be regulated by the target binding between Slit2 and its cognate receptors Robo1/2 [29]. A growing body of literature has shown that the activation of the Slit2/Robo1 axis can suppress the proliferation and invasion of multiple cancers, including cervical, colorectal and breast cancers [30–32]. Importantly, downregulated Slit2/Robo1 signaling is reported to suppress the tumor progression by regulating beta-catenin [22]. In this study, RA notably upregulated the expression of Slit2 and Robo1 in both Hela and c-33A cells, demonstrating the activation of this signal by RA. Existing study has shown that Slit2/Robo1 axis can lead to cell cycle arrest, which indirectly displays that RA promotes the cell cycle arrest by the activation of this signaling [29].

miRNAs are a group of small conserved ribonucleic acids with no protein-coding ability, and they are now believed to be closely related to the regulation of cell differentiation and cell cycle, thus playing an essential role in tumor progression [33]. Being one of the common miRNAs, miR-224-3p can promote the proliferation and metastasis of many malignant neoplasms [34]. Interestingly, miR-224-3p can be a target to settle cancer progression and poor prognosis as its expression in cervical cancer tissue is up-regulated and closely linked to HPV-activated status [16, 17]. The miRDB, an online database for predicting microRNA targets, predicted the target binding between miR-224-3p and Slit2, which was verified by luciferase activity reporter assay in this study. To further clarify the regulatory mechanism of RA in the progression of CC, miR-224-3p mimic was transfected into both Hela and c-33A cells, respectively. It was found that miR-224-3p overexpression dramatically restored the impact of RA treatment on invasion, migration, cell cycle arrest and apoptosis in Hela and c-33A cells. What’s more, significantly downregulated Slit2 and Robo1 expression levels were observed following CC cells transfected with miR-224-3p mimic.

Taken together, our findings demonstrated that RA inhibits proliferation, invasion and migration of cervical cancer cells via regulating miR-224-3p/Slit2/Robo1 signaling pathway, which indicates that RA can be an effective drug for CC therapies by targeting miR-224-3p and its downstream effectors. However, the concrete relationship between RA and miR-224-3p still needs further exploration, and the above-mentioned *in vivo* anti-tumor effects still need to be validated in future research. Additionally, whether these is a synergistic effect of RA on cervical cancer cell proliferation, invasion, migration and apoptosis when used in combination with any of the most commonly chemotherapeutic drugs used in cervical cancer will be investigated in the future, which is an another limitation of the present study.

## MATERIALS AND METHODS

### Cell culture and cell transfection

Hela and c-33A cells were purchased from American Type Culture Collection (ATCC). Cells were thawed and passaged in Dulbecco's modified Eagle's medium (DMEM) supplemented with 10% fetal bovine serum (FBS), 100 U/mL of penicillin, and 100 μg/mL of streptomycin in a humidified incubator with 5% CO_2_ at 37° C. RA, which was obtained from Glip Biotech (Chengdu, China), was used for treatment of the harvested cells. For transfection, cells were transfected with miR-224-3p mimic (5′-AAAAUGGUGCCCUAGUGACUACA-3′) or its negative control (miR-NC; 5′-UUCUCCGAACGUGUCACGUTT-3′) by using lipofectamine® 2000 reagent (Invitrogen; Thermo Fisher Scientific, Inc., Waltham, MA, USA) in accordance with the manufacturer’s guideline. And successful transfection was determined using reverse transcription-quantitative Polymerase Chain Reaction (RT-qPCR).

### Cell Counting Kit-8 (CCK-8) assay

Hela and c-33A cells were respectively collected at a number of approximately 5×10^3^ cells and seeded in 96-well plates. After cells were treated with different concentrations of RA (0, 1, 2, 4, 6, and 8 μM) for 24 h and 48 h respectively, cell viability was examined with CCK-8 detection kit (Dojindo Laboratories, Tokyo, Japan). For each well, 20 μl of CCK-8 solution was added and then cultured for 4 h. Finally, the absorbance at 450 nm by a microplate reader (Bio-Rad Laboratories, Richmond, CA, USA).

### Transwell assay

The invasive ability of Hela and c-33A cells was detected using 8-μm pore inserts coated with Matrigel (BD Biosciences, Franklin Lakes, NJ). Cells were harvested with serum-free DMEM and added to the upper compartment. DMEM containing 10% FBS was added to the lower compartment as a chemoattractant. After cells being cultivated for 24 h and 48 h in the incubator, the number of invading cells in five visual fields were counted under a light microscope and the mean was calculated.

### Wound healing assay

For wound healing assay, cells were incubated in a 6-well culture plate to achieve 80% confluence. Then, serum-free DMEM was utilized to incubate overnight prior to initiating the experiment. Cells were scratched horizontally with a 10 μl micropipette tip. The migrated cells were observed at 0 and 24 h after wounding using an inverted microscope (Olympus Corporation). Quantitative analysis of the wound healing area was performed using Image J software (National Institutes of Health).

### Flow cytometry for evaluation of cell apoptosis and cell cycle

Hela cells and c-33A cells were treated with RA at the dose of 4 μM for 24 h and 48 h, respectively. For the detection of cell apoptosis, the cells were then harvested and resuspended at a concentration of 1×10^6^ cells/ml after being washed by phosphate buffer saline (PBS) for three times. After 15 min of incubation in the dark, the cell suspensions (100 μl) containing annexin V-FITC (10 μl) and propidiom iodide (PI, 20 μg/ml) were then added to the labeled tube to detect cell apoptosis.

For the evaluation of cell cycle, cells were fixed with 70% ethanol for 12 h after three times of PBS washing to increase cell membrane penetrability. The cells were prepared with the Cell Cycle Analysis kit (Beyotime, Shanghai, China) according to the manufacturer’s protocol. After adding the binding buffer, RNase A, and PI, the cells were incubated at 37° C in the dark for 30 min and then analyzed by flow cytometry (Beckman Coulter, Inc.).

### Luciferase activity reporter assay

miRDB, an online database for predicting microRNA targets (http://mirdb.org/miRDB/) [35], was used to detect the target binding between miR-224-3p and Slit2. And luciferase activity reporter assays were executed for verifying this combination. Cells were co-transfected with miR-224-3p mimic or miR-NC and Slit2 3' UTR-wild type (WT) or Slit2 3' UTR-mutant (MUT) which were obtained from GenePharma (Shanghai, China). Lipofectamine 2000 (Invitrogen, Carlsbad) was applied for the transfection procedure. The Dual Luciferase Reporter Assay kit (Promega, Madison, WI, USA) was employed to evaluate the relative luciferase signals to evaluate the targeted regulatory effect of miR-224-3p on Slit2.

### RT-qPCR

Total RNA was respectively extracted from Hela cells and c-33A cells with Trizol reagent (Invitrogen). Complementary DNA (cDNA) was synthesized using the PrimeScript RT Reagent Kit (Takara, Japan) for the PCR template. PCR then was performed with 2 μg cDNA as the templet using Power SYBR Master Mix (Applied Biosystems, Foster, USA) on the ABI 7500 PCR system (Applied Biosystems). The primers of miR-224-3p and control U6 were synthesized by GenePharma (Shanghai, China). The cycling conditions were used as follows: denaturation at 95° C for 10 min, 40 cycles at 95° C for 10 s, and amplification at 60° C for 34 s. U6 was considered as an internal control.

### Western blot analysis

Cells were harvested to extract protein by lysis from RIPA lysis buffer (Beyotime, Shanghai, China). The bicinchoninic acid protein assay kit (Pierce, Rockford, IL, USA) was used for the detection of protein concentrations. Sodium dodecyl sulfate-poly acrylamide gel electrophoresis (SDS-PAGE) gel was used to resolve the proteins, and then the proteins were transferred to PVDF membranes. Next, the membranes were blocked with 5% non-fat milk and later incubated with primary antibodies of apoptosis-associated proteins (Bcl-2, Bax and cleaved caspase-3), cell cycle-related proteins (cyclinD1, cyclinD2, cyclin-dependent kinase 2 (CDK2), p21and p27), and migration-related proteins (matrix metalloproteinase (MMP)-2 and MMP-9). Finally, after horseradish peroxidase-labelled secondary antibody were incubated on the membranes for 1 h, these bands were visualized using Odyssey Infrared Imaging Scanner (LI-COR Biosciences). Protein expression was quantified using Image J software. GAPDH was serves as a loading control.

### Statistical analysis

Statistical analysis was performed with GraphPad Prism 6 (GraphPad Software, Inc., La Jolla, CA, USA). All data were expressed as mean ± standard deviation. Student’s *t*-test was used for comparison between two groups. An analysis of variance (ANOVA) with Tukey's post hoc test was used for comparison among multiple groups. Statistical significance was established when *P* < 0.05.

### Availability of data and material

The analyzed data sets generated during the study are available from the corresponding author on reasonable request.
